# Electrocardiographic abnormalities in treatment-naïve HIV subjects in south-east Nigeria

**DOI:** 10.5830/CVJA-2017-013

**Published:** 2017

**Authors:** Innocent Chukwuemeka Okoye, Ernest Ndukaife Anyabolu

**Affiliations:** Chukwuemeka Odumegwu Ojukwu University, Awka, Nigeria; Imo State University, Orlu, Nigeria

**Keywords:** ECG, cardiac abnormalities, non-cardiac ECG abnormalities, HIV, Enugu, Nigeria

## Abstract

**Background:**

Cardiac complications of human immunodeficiency virus (HIV) infection are important causes of morbidity and mortality. We set out to determine the electrocardiographic (ECG) abnormalities in treatment-naïve HIV-positive patients in Enugu, south-east Nigeria.

**Methods:**

This was a cross-sectional study involving 250 HIV-positive and 200 HIV-negative subjects. Demographic and anthropometric data, relevant investigations and ECG results were compared between the groups.

**Results:**

An abnormal ECG was present in 70% of the HIV-positive patients, sinus bradycardia in 64%, QTC prolongation in 48%, T-wave inversion in 21.6%, Wolf–Parkinson– White syndrome in 0.8%, abnormal P waves in 12.8%, 1st degree heart block in 2.4%, ST depression in 30%, and left-axis deviation in 1.6%. Underweight was associated with ECG abnormalities (p = 0.001). The HIV-positive patients had more ECG abnormalities than the HIV-negative subjects (p = 0.001).

**Conclusion:**

Electrocardiographic abnormalities were common in treatment-naïve HIV-positive patients in Enugu, Nigeria. The 70% prevalence of ECG abnormalities in treatment-naïve HIV-positive patients was high. There is a need to evaluate HIV-positive patients at onset for cardiac and non-cardiac abnormalities detectable by ECG.

## Introduction

About 70% of the world’s human immunodeficiency virus (HIV)-infected persons live in sub-Saharan Africa.^1^ In Nigeria, the prevalence of HIV infection is 3.7%.^1^

Cardiac lesions have been reported in HIV-positive patients.^2^,^3^ Mpiko and Hakim showed that among the peculiar features of HIV-related cardiovascular disorders in sub-Saharan Africans, pericardial effusion may be the first manifestation of the illness. Prevalent infectious diseases were seemingly mirrored by the aetiology of cardiac disease, and specific cardiovascular disorders were associated with HIV infection.^4^

Electrocardiographic (ECG) and echocardiography abnormalities have been demonstrated in HIV-positive patients.^2^,^3^,^5^-^7^ The ECG abnormalities documented include arrhythmias, low-voltage QRS complexes, non-specific ST-segment and T-wave changes, poor R-wave progression, right bundle branch block, axis deviations, enlargement of various heart chambers and QTC prolongation.^8^,^9^ The manifestations of HIV infection in organs other than the heart mask the clinical evidence of cardiac disease in these subjects.^10^-^13^

Electrocardiography is effectively used to detect cardiac diseases.^14^ Cardiac diseases attributable to HIV infection are of public health importance because they are usually silent, yet have the potential to cause high mortality rates.

There is a paucity of studies on ECG abnormalities in treatment-naïve HIV-positive patients emanating from southeast Nigeria, prompting us to embark on this study. This will help in identifying HIV-positive patients who may have cardiac and non-cardiac illnesses, detectable by ECG, with a view to instituting appropriate early interventions to whittle down adverse outcomes in this group of patients. In addition, ECG is cheap and readily available.

## Methods

This was a cross-sectional study conducted at the University of Nigeria Teaching Hospital (UNTH), Enugu, Nigeria, between September and December 2015. The study subjects consisted of 250 treatment-naïve HIV-positive patients and 200 HIV-negative subjects as controls, consecutively recruited from an HIV clinic and the medical wards of the hospital.

Inclusion criteria were subjects with confirmed HIV-positive tests, aged 15 years and older. Those subjects who had hypertension, pre-morbid cardiac diseases, a history of cigarette smoking and significant alcohol use, those on medications known to affect the cardiovascular system, pregnant women and puerperal women up to three months, those with diabetes mellitus, acromegaly or thyrotoxicosis were all excluded from the study.

Informed consent was obtained from all the subjects who participated in this study. The ethics committee of UNTH approved the study.

Demographic and other relevant data were obtained with the help of a questionnaire. Physical examination was done on each subject. Anthropometric data were obtained: height (m) and weight (kg). Body mass index (BMI) was recorded as weight/height2 (kg/m2). Blood pressure (mmHg) was measured, systolic blood pressure (SBP) at Korotkoff phase 1 and diastolic blood pressure (DBP) at phase 5 or at phase 4 when the differences between phase 4 and 5 were more than 10–20 mmHg. Body temperature (°C) was taken and evidence of cardiac decompensation determined.

Every subject had a 12-lead surface ECG, using a long-lead V1 complex as a rhythm strip with three-channel automated Schiller ECG machine (Switzerland) AT-1. Other investigations done were standard postero-anterior chest X-rays for detecting occult cardiopulmonary lesions and for estimating cardiothoracic ratio (CTR), serum urea, creatinine, electrolyte and calcium levels, and CD4 (clusters of differentiation 4) cell count.

## Statistical analysis

The data were analysed using SPSS version 15.0. Descriptive statistics were used to determine the mean values of the variables and the median value of the CD4 cell counts in the study population. Chi-squared analysis was used for testing for significant differences between proportions and frequencies, while the Student’s t-test was used to compare continuous variables between the treatment-naïve HIV-positive patients and HIV-negative controls. A p-value < 0.05 was taken as statistically significant.

## Results

Out of 250 treatment-naïve HIV-positive subjects studied, 124 (49.6%) were female and 126 (50.4%) were male. Out of 200 HIV-negative control subjects, 107 (53.5%) were female and 93 (46.5%) were male.

The HIV-positive subjects comprised Igbo 232 (92.6%), Hausa seven (3.1%), Igala five (2.0%) and other groups six (2.3%). By contrast, the HIV-negative control subjects were made up of Igbo 178 (89.0%), Hausa six (2.9%), Igala six (2.9%) and other groups 10 (5.2%). The majority of the study subjects (94.5%) was Christian.

All the subjects were in the age range of 15–60 years. The mean age of the treatment-naïve HIV-positive subjects was 34.89 ± 10.58 years and the controls was 36.04 ± 12.61 years. There was no significant difference between the ages of the two groups (p = 0.146).

Table 1 shows the descriptive statistics of the study subjects. The mean BMI, SBP and DBP were significantly lower in the treatment-naïve HIV-positive subjects than in the HIV-negative control subjects. Most (70%) of the HIV-positive subjects had a BMI < 18.5 kg/m^2^ (underweight). The mean body temperature was significantly higher in the HIV-positive subjects than in the HIV-negative subjects.

**Table 1:** Descriptive statistics of the study and control groups

*Parameters*	*Group*	*Sample size*	*Mean*	*SD*	*p-value*
Age (years)	HIV+	250	34.89	10.58	0.146
	HIV–	200	36.04	12.61	
BMI (kg/m^2^)	HIV+	250	20.05	1.522	< 0.001
	HIV–	200	22.77	2.663	
Sitting SBP (mmHg)	HIV+	250	104.2	10.27	< 0.001
	HIV-	200	119.1	9.97	
Sitting DBP (mmHg)	HIV+	250	76.6	4.747	0.848
	HIV–	200	76.9	4.637	
Standing SBP (mmHg)	HIV+	250	98.96	10.63	< 0.001
	HIV–	200	115.0	5.84	
Standing DBP (mmHg)	HIV+	250	77.64	5.267	< 0.001
	HIV–	200	75.25	5.203	
Temperature (°C)	HIV+	250	37.54	0.60	< 0.001
	HIV–	200	36.67	0.45	

SD = standard deviation, BMI = body mass index, SBP = systolic blood pressure, DBP = diastolic blood pressure.

The mean serum albumin level (22.14 g/l) was low in the treatment-naïve HIV-positive subjects (Table 2). Low CD4 cell counts (< 200 cells/ml) were present in 75 (30%) of HIV-positive subjects.

**Table 2:** Laboratory characteristics of the study population (n = 250)

*Parameters*	*HIV-positive patients (mean + SD)*	*Non-HIV-negative controls (mean + SD)*
Na^+^ (mmol/l)	131.5 ± 4.1	134.2 ± 4.2
K^+^ (mmol/l)	3.2 ± 0.2	4.2 ± 0.3
HCO_3_^+^ (mmol/l)	22.2 ± 0.5	23.3 ± 1.1
Urea (mmol/l)	5.8 ± 0.3	6.1 ± 0.6
Ca^2+^ (mmol/l)	2.1 ± 0.1	2.4 ± 0.2
Albumin (g/l)	22.1 ± 0.8	3.5 ± 0.8
CD4 cells/ml (median)	390	645

SD = standard deviation, Na^+^ = serum sodium, K^+^ = serum potassium, HCO_3_^+^ = serum bicarbonate, Ca^2+^ = serum calcium.

All the subjects, both treatment-naïve and HIV-negative, had normal cardiac apex and normal chest X-rays.

An abnormal ECG was present in 175 (70%) of the 250 treatment-naïve HIV-positive subjects, and 70 (35%) of the 200 HIV-negative subjects. Table 3 shows the various ECG abnormalities in the study population. Sinus tachycardia was present in 160 (64%) of the HIV-positive subjects, prolonged QTC in 120 (48%), ST depression in 75 (30%) and T-wave inversion in 54 (21.6%). Table 3 shows all the ECG abnormalities.

**Table 3:** Various ECG abnormalities in the study population

*ECG abnormalities*	*Treatment-naïve HIV+ patients, n (%)*	*HIV-negative subjects, n (%)*	*p-value*
Sinus tachycardia	160 (64)	24 (12)	< 0.001
Sinus bradycardia	2 (0.8)	14 (7)	0.201
Prolonged QTC	120 (48)	16 (8)	< 0.001
Shortened PR interval (WPW)	2 (0.8)	0	< 0.001
ST depression	75 (30)	4 (2)	< 0.001
T-wave inversion	54 (21.6)	16 (8)	0.011
Left-axis deviation	4 (1.6)	4 (2)	< 0.001
Left atrial enlargement	32 (12.8)	16 (8)	0.048
1st degree heart block	6 (2.4)	0	0.005
Left anterior hemiblock	2 (0.8)	0	0.024
Incomplete RBBB	4 (1.6)	0	0.012
RVH	2 (0.8)	4 (2)	0.684
LVH	35 (14)	20 (10)	0.044
Ventricular ectopics	10 (4)	2 (1)	0.019
Atrial ectopics	2 (0.8)	0	0.041
Low QRS in all leads	10 (4)	0	0.068
Low QRS in limb leads	8 (3.2)	0	0.074

RBBB = right bundle branch block, RVH = right ventricular hypertrophy, LVH = left ventricular hypertrophy.

Comparison of the mean ECG parameters between the study groups is shown in Table 4. The mean heart rate, axis, PR interval and QTC were significantly higher in treatmentnaïve HIV-positive subjects than in the HIV-negative subjects. Conversely, the mean QRS duration did not differ significantly between the two groups. All the subjects in the study were in sinus rhythm.

**Table 4:** Comparison of ECG parameters between treatment-naïve HIV-positive patients and HIV-negative subjects

*Parameter*	*Group*	*Sample size*	*Mean*	*SD*	*p-value*
Heart rate (beats/min)	HIV+	250	99.6	11.53	< 0.001
	HIV–	200	84.36	5.35	
Axis (degrees)	HIV+	250	45.64	61.23	< 0.001
	HIV–	200	31.38	11.78	
PR interval (s)	HIV+	250	0.16	0.03	< 0.001
	HIV–	200	0.14	0.01	
QRS duration (s)	HIV+	250	0.07	0.04	0.068
	HIV–	200	0.06	0.01	
QTC	HIV+	250	0.44	0.03	< 0.001
	HIV–	200	0.39	0.01	

SD = standard deviation

Out of the 250 HIV-positive patients, 160 (64.0%) had tachycardia, while 24 (12.0%) of the 200 HIV-negative subjects had tachycardia. This difference was statistically significant (p < 0.001). However, among the 160 HIV-positive patients with tachycardia, 40 (25.0%) had fever, while 120 (75.0%) did not have fever. By contrast, among the 24 HIV-negative subjects with tachycardia, 19 (79.2%) had fever while five (20.8%) did not. When fever was excluded, the prevalence of tachycardia was significantly higher in the HIV-positive patients than the HIV-negative controls.

The mean axis was 45.64 ± 6.22° in the treatment-naïve HIV-positive subjects. One subject has left-axis deviation of –30° and three had left-axis deviation of –60°, while none had right axis deviation.

Thirty-two (12.8%) of the 250 treatment-naïve HIV-positive subjects had abnormal P waves with P mitral in lead II with or without biphasic P wave in lead VI. By contrast, this was seen in 16 (8.0%) of the HIV-negative subjects.

The mean PR interval was 0.16 ± 0.03 seconds in the treatment-naïve HIV-positive patients. Six subjects had prolonged PR intervals (1st degree heart block), while two had shortened PR intervals with associated delta waves and widened QRS complexes, evidence of Wolf–Parkinson–White (WPW) syndrome. No abnormal PR interval was seen in the HIV-negative subjects.

The mean QRS duration was 0.07 ± 0.01 seconds. The two subjects with QRS > 0.01 seconds were the same patients that had WPW syndrome stated above.

Thirty per cent of the subjects had ST depression in two or more leads. There was no ST elevation in either group.

T-wave inversion was present in 54 (21.6%) of the 250 treatment-naïve HIV-positive subjects. Sixteen (8%) of the HIV-negative subjects had inverted T waves.

The mean QTC interval was 0.44 ± 0.03 seconds in the treatment-naïve HIV-positive subjects. Of these, 48% had prolonged QTC intervals, compared to 8% of the HIV-negative subjects with same QTC intervals. This difference was significant (p < 0.001). Furthermore, among the subjects without hypocalcaemia, QTC prolongation was observed more often in the HIV-positive patients than the HIV-negative controls. This difference was statistically significant (p < 0.001).

The mean serum urea level in the study group was 5.803 ± 0.227 mmol/l. Sixty-four (25.6%) of the 250 HIV-positive patients had a serum urea level > 6.5 mmol/l,

The mean serum potassium level was 3.168 ± 0.167 mmol/l. Out of the 250 treatment-naïve HIV-positive patients, 150 (60%) had serum potassium levels < 3.5mmol/l, while none had serum potassium levels ≥ 5.5 mmol/l.

The mean serum calcium level in the study group was 2.06 ± 0.133 mmol/l. Hypocalcaemia was observed in 25% of the treatment-naïve HIV-positive patients. Forty-six per cent of the patients had hypoalbuminaemia (albumin < 2.8 g/dl).

One hundred and twenty (48%) of the treatment-naïve HIV-positive patients had diarrhoea.

## Discussion

The prevalence of ECG abnormalities in HIV-positive patients at UNTH (70%) was significantly higher, compared to the 35% in HIV-negative subjects. This was similar to the 86% reported by Mounodji et al. in Chad,^8^ but higher than the 53% seen by Levy et al. in Washington, USA,^15^ and the 55% reported by Herst et al. in Ontario, Canada.^16^ These observed differences could be explained by the differences in the study design; the study population was 250 in our study, 32 in the study by Mounodji et al., and 21 in the study by Levy et al. In addition, only patients with Kaposi sarcoma were evaluated in the latter study. Cardiac abnormalities in HIV-positive patients were reported based on either autopsy findings or more advanced cardiac investigations such as echocardiography and Doppler studies. Nevertheless, the prevalence rate of ECG abnormalities in our study was within the general prevalence rate of 28 and 73% documented in some studies.^3^,^8^,^16^,^17^

The cachectic heart, a clinical pathological and ECG entity seen in chronic debilitating diseases, has been reported in HIV-positive patients.^18^-^20^ In our study, 48% of the treatment-naïve HIV-positive patients had low BMI, and ECG abnormalities were found in 80% of those patients with a low BMI (underweight). We also demonstrated that BMI had a significant effect on ECG abnormalities (p < 0.001). This shows that the ECG abnormalities observed in our study may have been contributed to by the low BMI, among various mechanisms elucidated in the pathogenesis.^21^,^22^

The prevalence of the various ECG abnormalities seen in HIV-positive patients at UNTH was in order of frequency: sinus tachycardia (64%), prolonged QTC (48%) and T-wave inversion (21.6%). This compares favourably with a similar study by Sani in Jos, Nigeria.^23^ Mounodji et al. reported sinus tachycardia in 31% of 55 patients studied in Chad.^7^ Sinus tachycardia was the second commonest ECG abnormality after low-voltage QRS complexes in their series.

Heart rate is known to increase with a rise in body temperature.^24^ When fever was excluded in this study and tachycardia was compared between the treatment-naïve HIV-positive patients and the HIV-negative controls, the difference was statistically significant (p < 0.001). This shows that the tachycardia we noted could not be explained by pyrexia, which some of the patients had. Unexplained fever is a feature of myocarditis, and myocarditis can be caused by HIV infection.^4^

Some workers, however, view tachycardia as being due to excessive sympathetic stimulation, which could be caused by autonomic imbalance or stimulation of beta-receptors by the gp 120 protein of HIV.^25^ Emotion may be an additional contributory factor.^26^ However, dehydration and underweight, measures of malnutrition in developing countries, could also explain, in part, the sinus tachycardia observed in our study.

QTC prolongation with no known cause was reported in 69% of AIDS patients studied by Kocheril et al.^27^ This rate is lower than the 48% found in our study. In their study, AIDS patients were evaluated, while in ours, it was treatment-naïve HIV-positive patients. This could, perhaps, account for the differences in the prevalence observed.

Although QTC prolongation and torsades de pointes are known to occur with pentamidine therapy,^28^ none of the patients in our study was on pentamidine. Our study also showed that among subjects who did not have hypocalcaemia, QTC prolongation was observed more in HIV-positive patients than in HIV-negative controls. The QTC prolongation in this study may therefore have been due to non-specific ECG abnormalities found in HIV-positive patients.^29^

In the present study, 30% of the treatment-naïve HIV-positive patients had ST-segment depression while 21.6% had T-wave inversion. Non-specific ST and T-wave changes on ECG are seen in AIDS patients, caused either by pericardial disease or dilated cardiomyopathy, which are known to occur in HIV-positive patients.^29^,^30^

Low-voltage QRS complexes in all the leads may have been due to pericardial effusion. Pericardial effusion has been reported as the commonest cardiac manifestation of HIV infection.^15^ Low QRS complexes in only the limb leads are a feature of dilated cardiomyopathy.

Other ECG abnormalities found in this study were atrial and ventricular ectopics. Although the prevalence of these ectopics did not differ significantly between the treatmentnaïve HIV-positive patients and the HIV-negative subjects, these premature contractions could be explained by possible myocarditis, abnormalities in the conduction system and derangement of the autonomic nervous system, which are all known to occur in these patients.

## Conclusion

Cardiac and non-cardiac abnormalities, detectable by ECG, were common in treatment-naïve HIV-positive patients in Enugu, Nigeria. The 70% prevalence of ECG abnormalities in treatmentnaïve HIV-positive patients was high. There is a need to evaluate this group of patients at onset for cardiac and non-cardiac abnormalities detectable by ECG. Further research could explore how some of these abnormalities are generated in HIV infection.
